# Therapeutic potential of flower fragrance diffusion on heart rate variability among hospitalized patients

**DOI:** 10.6026/973206300214324

**Published:** 2025-12-15

**Authors:** Gurleen Kaur, Yogesh Kumar, Nirangjhana S, Vishnu Shankar Ojha, Ratandeep Biswas

**Affiliations:** 1 All India Institute of Medical Sciences Patna, India; 2 Department of Physiology, All India Institute of Medical Sciences Patna, India

**Keywords:** Aromatherapy, floral fragrance, heart rate variability, hedonic perception

## Abstract

Heart Rate Variability (HRV) is a non-invasive measure of autonomic nervous system activity and may be influenced by environmental odors. Therefore, it is of interest to evaluate the effect of flower fragrance diffusion on HRV in hospitalized patients. Thirty adults admitted to obstetrics and chemotherapy wards underwent baseline HRV assessment, 60-minute flower fragrance exposure and post-intervention HRV recording. Hedonic responses were rated on an 11-point scale pre- and post-exposure. Fragrance diffusion significantly increased HRV parameters including SDNN (p<0.05), rMSSD (p<0.01) and pNN50 (p<0.001), with improved hedonic ratings (p<0.001). Hedonic responses were positively correlated with HRV (r=0.42, p<0.01), suggesting flower fragrance diffusion favorably modulates HRV and emotional responses, suggesting potential benefit as a supportive intervention in clinical wards.

## Background:

Heart rate variability (HRV) is a well-established non-invasive index for assessing autonomic nervous system (ANS) regulation of the sinoatrial node, derived from fluctuations in successive R-R intervals on the electrocardiogram [1]. HRV reflects dynamic interactions between the ANS and cardiac function during processes such as exercise, sleep and respiration [2]. High HRV is generally associated with better cardiovascular health and adaptive stress response, whereas reduced HRV has been linked to stress, illness and impaired autonomic regulation [3, 4]. The olfactory system strongly influences emotional, cognitive and physiological responses [5]. Fragrances can evoke emotional experiences [6] and modulate ANS activity, particularly HRV, which is a marker of autonomic balance [7]. Aromatherapy, defined as the therapeutic use of plant-derived fragrances, has long been applied to promote relaxation and well-being [8]. Recent studies indicate that lavender and citrus essential oils reduce anxiety [9], improve subjective well-being [10], enhance sleep quality [11, 12] and support cognitive function [13]. Furthermore, fragrance exposure has been shown to increase parasympathetic activity while reducing sympathetic dominance in healthy adults [14]. Despite this evidence, the application of fragrance-based interventions in hospital environments remains limited. Odors from antiseptics and disinfectants in clinical wards may contribute to stress and discomfort [15]. Few studies have systematically examined the effects of floral fragrance diffusion on HRV and patient-reported hedonic responses in hospitalized patients. Therefore, it is of interest to evaluate the effects of 60-minute flower fragrance diffusion on HRV in patients admitted to obstetrics and chemotherapy wards and to explore correlations between hedonic responses and HRV changes.

## Materials and Methods:

This pre-post study was conducted at the Department of Physiology of AIIMS, Patna, after receiving ethical clearance from the Institutional Ethics Committee (approval number: AIIMS/PAT/IEC/2020/623). Study duration was six months. A total of thirty adult participants, aged between 18 to 60 years, were enrolled after obtaining written informed consent. Sample size was calculated based on the ability to detect a moderate effect size (Cohen's d = 0.6) in HRV parameters between pre and post fragrance exposure, with a significance level of 0.05 and power of 80%. Using G Power software for paired-sample t-test, the required sample size was determined to be 24. To account for potential dropouts or data loss, a total of 30 participants were recruited. Participants were recruited from the Obstetrics and Chemotherapy wards using convenience sampling, with fifteen participants from each ward. The inclusion criteria were: Patients admitted in the Obstetrics or Chemotherapy wards, no history of anosmia or parosmia, free from any ENT disorders affecting olfactory function. Exclusion criteria included pregnancy, severe respiratory conditions and known allergies to floral scents. After obtaining written informed consent, demographic details including name, age, sex, address, weight and height were recorded for each participant.

## Baseline assessment:

Participants were instructed to abstain from caffeine, nicotine and alcohol for at least 4 hours prior to the assessment. Baseline HRV recordings were conducted using the AD Instrument PowerLab system (PowerLab 8/35, ADInstruments, Australia). ECG electrodes were placed on the right arm (negative), left arm (positive) and right leg (ground). Participants rested in a supine position for five minutes before the recording began. The following HRV parameters were recorded: SDNN (Standard Deviation of NN intervals), rMSSD (Root Mean Square of Successive Differences), pNN50 (Proportion of NN50 divided by total number of NNs), LF (Low Frequency power), HF (High Frequency power) and LF/HF ratio. These parameters were chosen based on their established relevance in assessing autonomic nervous system function and their sensitivity to short-term changes. Additionally, participants were asked to rate the ambient odor in the ward on an 11-point hedonic scale ranging from -5 (highly unpleasant) to +5 (highly pleasant), with 0 representing a neutral response.

## Fragrance intervention:

Following the baseline assessment, participants were exposed to a flower fragrance for 60 minutes. The fragrance used was a blend of lavender (Lavandula angustifolia) and jasmine (Jasminum officinale) essential oils, chosen for their well-documented calming and mood-enhancing properties. The fragrance was diffused using an ultrasonic aromatherapy diffuser (Godrej aer Matic Room fresheners automatic machine) placed 2 meters away from the participant's bed. Room conditions were standardized across all sessions with temperature: 22-24°C, relative humidity: 40-60% and air exchange rate: 6-8 air changes per hour.

## Post-Intervention assessment:

Immediately following the 60-minute fragrance exposure, HRV parameters were recorded again using the same protocol as the baseline assessment. Participants also provided post-exposure hedonic ratings of the fragrance.

## Statistical analysis:

Data analysis was performed using SPSS IBM software version 25. The Shapiro-Wilk test was used to assess the normality of variables. Normally distributed data are presented as mean ± standard deviation (SD), while variables with skewed distribution are presented as median (interquartile range). To compare HRV parameters pre- and post-exposure, paired t-tests were used for normally distributed variables and Wilcoxon Signed Rank tests were used for variables with skewed distribution. Spearman's correlation coefficient was calculated to assess the relationship between hedonic responses and HRV parameters. p-value < 0.05 (2-tailed) was considered to be statistically significant.

## Results:

A total of 30 participants were included in the study, with 15 each from the Chemotherapy and Obstetrics wards. The baseline characteristics of participants are summarized in Table 1 (see PDF) and the gender distribution is displayed in presented in Figure 1 (see PDF). Changes in HRV parameters before and after exposure to the floral fragrance are presented in Table 2 (see PDF) and Table 3 (see PDF) outlines the hedonic ratings of ambient odor before and after fragrance exposure. Both chemotherapy and obstetrics wards showed significant improvements in hedonic scores following flower fragrance exposure (p = 0.001 for both wards). The median scores decreased, indicating a shift towards more pleasant perceptions of the ambient odor. The correlation between hedonic scores and HRV parameters are presented in Table 4 (see PDF).

## Discussion:

The findings of this study indicate that flower fragrance has a positive impact on heart rate variability (HRV) in patients admitted to obstetrics and chemotherapy wards. In the chemotherapy ward, significant changes were observed in several HRV parameters following flower fragrance exposure. Specifically, there was a significant reduction in LF power (p = 0.047) and LF/HF ratio (p = 0.013). These changes suggest an overall reduction in sympathetic activity, indicating a more relaxed state. In the obstetrics ward, no statistically significant changes were observed in HRV parameters. However, there was a trend towards increased HF power and decreased LF/HF ratio, suggesting a possible shift towards parasympathetic dominance. The positive effect of flower fragrance on HRV observed in this study is consistent with earlier research on the physiological impacts of aromatherapy. Recent work and reviews from Sattayakhom *et al.* 2023 and Wang *et al.* 2023 [16, 17] have likewise shown that inhalation of essential oils such as lavender and sweet orange reduces heart rate and blood pressure and shifts sympathovagal balance toward parasympathetic dominance (lower LF/HF). Similar to our findings, these studies reported that pleasant fragrances could lower the LF/HF ratio, a key marker of reduced sympathetic dominance. In the chemotherapy ward, significant positive correlations were found between hedonic scores and pNN50 (r = 0.416, p = 0.011). These correlations suggest that as hedonic scores improved (became more pleasant); there was an increase in parasympathetic activity. These results align with previous research that has demonstrated the influence of pleasant olfactory stimuli on autonomic nervous system activity, particularly in enhancing parasympathetic tone and reducing sympathetic activity [16, 17]. In the context of patient comfort and well-being, the results corroborate the work of Fukui *et al.* [18], who explored the neuroendocrinological effects of saffron odor on mood and autonomic responses. They found that the fragrance had a calming effect, reducing stress and improving emotional well-being, which aligns with the significant positive hedonic responses recorded in our study participants post-exposure. Interestingly, while our study shows a marked improvement in HRV parameters in chemotherapy patients, the obstetrics ward results were more variable. This divergence might be attributed to differences in baseline stress levels or emotional states between the two patient groups, as supported by studies like those of Xie *et al.* [19], which highlighted the differential impact of olfactory stimuli on various patient demographics. The differential responses observed between obstetrics and chemotherapy wards highlight the need for tailored approaches in fragrance therapy. The significant correlation between hedonic responses to fragrance and HRV parameters further emphasizes the role of olfactory stimuli in modulating emotional and physiological states. The findings suggest that incorporating use of fragrances into clinical settings, particularly in wards where patients experience high levels of stress or discomfort, could be a simple yet effective strategy to enhance patient well-being. Given the evidence, it may be prudent to integrate such interventions more widely, particularly in palliative care and long-term hospital stays, where patient comfort and quality of life are paramount. Future research could explore optimizing fragrance types and exposure times, as well as investigating the long-term effects of such interventions on patient outcomes.

## Limitations and future research:

While the study provides valuable insights, it is important to consider the limitations, including the small sample size and the specific patient populations studied. Larger studies encompassing a broader range of patient demographics would help validate these findings. Additionally, further research could investigate the mechanisms underlying the observed effects, possibly through neuroimaging studies to explore how olfactory stimuli influence brain regions associated with autonomic regulation. Future research should focus on optimizing fragrance types, exposure durations and application methods for specific patient populations. Additionally, long-term studies are needed to assess the sustained effects of fragrance interventions on patient outcomes and quality of life.

## Conclusion:

We show the therapeutic potential of fragrance diffusion in clinical settings. By improving heart rate variability and eliciting positive emotional responses, flower fragrances could serve as a non-invasive and cost-effective approach to enhance patient care, particularly in environments where traditional medical interventions may not fully address the emotional and psychological needs of patients.

## Financial support and sponsorship:

ICMR-STS.
